# Artificial intelligence applications for assessing ultra-processed food consumption: a scoping review

**DOI:** 10.1017/S000711452510593X

**Published:** 2026-02-28

**Authors:** Jessica L. Campbell, Grant Schofield, Hannah R. Tiedt, Caryn Zinn

**Affiliations:** 1 Human Potential Centre, Faculty of Health and Environmental Sciences, Auckland University of Technology, Auckland 1142, New Zealand; 2 Sports Performance Research Institute New Zealand, Auckland University of Technology, Auckland 1142, New Zealand

**Keywords:** Artificial intelligence, Ultra-processed foods, NOVA classification, Machine learning, Food processing, Scoping review

## Abstract

Ultra-processed foods (UPF), defined using frameworks such as NOVA, are increasingly linked to adverse health outcomes, driving interest in ways to identify and monitor their consumption. Artificial intelligence (AI) offers potential, yet its application in classifying UPF remains underexamined. To address this gap, we conducted a scoping review mapping how AI has been used, focusing on techniques, input data, classification frameworks, accuracy and application. Studies were eligible if peer-reviewed, published in English (2015–2025), and they applied AI approaches to assess or classify UPF using recognised or study-specific frameworks. A systematic search in May 2025 across PubMed, Scopus, Medline and CINAHL identified 954 unique records with eight ultimately meeting the inclusion criteria; one additional study was added in October following an updated search after peer review. Records were independently screened and extracted by two reviewers. Extracted data covered AI methods, input types, frameworks, outputs, validation and context. Studies used diverse techniques, including random forest classifiers, large language models and rule-based systems, applied across various contexts. Four studies explored practical settings: two assessed consumption or purchasing behaviours, and two developed substitution tools for healthier options. All relied on NOVA or modified versions to categorise processing. Several studies reported predictive accuracy, with F1 scores from 0·86 to 0·98, while another showed alignment between clusters and NOVA categories. Findings highlight the potential of AI tools to improve dietary monitoring and the need for further development of real-time methods and validation to support public health.

Ultra-processed foods (UPF), as defined by the NOVA classification system, are industrial formulations primarily composed of extracted food substances, often containing additives and minimal whole foods^(1)^. Characterised by hyper-palatability, convenience and high levels of added sugars, saturated fats and Na, these foods now dominate food supplies in high-income countries and are increasingly common in middle-income settings^(2–4)^. In many populations, they account for over half of dietary energy intake, particularly in the diets of adolescents and socio-economically disadvantaged groups^(3,5,6)^. High consumption of UPF has been associated with diet-related chronic diseases, including obesity, type 2 diabetes, CVD, cancer, respiratory disease and mental ill health, and is also associated with increased all-cause mortality^(7–13)^. Prompted by these health impacts, public awareness is growing, with several countries integrating UPF reduction into dietary guidelines^(14)^. Research on this topic has also rapidly expanded, with over 1300 articles published and a steep rise in systematic reviews since 2012^(15)^.

NOVA^(1)^ is the most widely used framework for classifying food processing levels, but other systems such as International Agency for Research on Cancer (IARC)^(16)^, International Food Information Council (IFIC)^(17)^, SIGA^(18)^ and HISS (the Human Interference Scoring System)^(19)^ use varying categories and criteria. However, despite the plethora of classification systems and associated literature, assessing UPF intake remains challenging; classifying foods by processing level is complex, with variability in application even among trained coders and public reliance on heuristics that can often miss nuanced criteria^(20,21)^. Recent comparative analyses have shown that applying different classification systems (e.g. NOVA, IFIC, The International Food Policy Research Institute (IFPRI), IARC and University of North Carolina (UNC)) to the same dietary datasets can yield widely divergent estimates of UPF intake^(22)^, highlighting the lack of harmonisation across frameworks. Traditional dietary assessment tools, such as 24-h recalls and FFQ, remain validated and widely used for estimating nutrient and energy intakes in population studies^(23–25)^. However, most were developed during a reductionist era focused on nutrient composition rather than the degree of food processing and therefore are not well suited to capturing processing dimensions or distinguishing UPF from minimally processed foods. As a result, classification by processing level often requires subjective coding and can vary considerably among researchers. Artificial intelligence (AI) approaches offer the potential to complement these established methods by automating classification, reducing coder variability and enabling scalable, data-driven assessment of food processing characteristics.

AI is increasingly being explored in the context of nutrition, particularly to enhance dietary assessment and support healthier dietary choices^(26)^. AI techniques such as computer vision (which analyses food images) and natural language processing (NLP, which processes text like ingredient lists) enable automated, scalable and accurate analysis of dietary intake data^(27,28)^. These AI approaches show promise for classifying UPF supporting real-time monitoring in clinical and public health settings.

Recent reviews have mapped AI applications in nutrition, including food detection, calorie tracking, nutrient profiling, dietary pattern analysis and scope for specific applications such as weight loss^(26–31)^. However, to date, no review has focused on the application of AI methods specifically for addressing UPF. Given the global scale of UPF consumption and its profound health, economic and environmental consequences^(8)^, there is an urgent need to evaluate AI-driven approaches to assess UPF intake and support healthier dietary choices.

## Objective

This scoping review aims to map the use of AI in assessing and classifying UPF consumption, examining AI methods, alignment with food processing frameworks (e.g. NOVA or other systems), accuracy and validation, and applications across clinical, research and public health settings. It seeks to identify current capabilities, limitations and opportunities for AI-driven UPF monitoring. It aims to answer the question, ‘What are the current uses of AI for assessing and classifying UPF consumption, focusing on methods, accuracy, and application scope?’

## Methods

### Study design

This scoping review was conducted to map the literature on AI-driven research for the recognition and assessment of UPF. The review followed the Preferred Reporting Items for Systematic Reviews and Meta-Analyses (PRISMA) Extension for Scoping Reviews (PRISMA-ScR) guidelines to ensure systematic and transparent reporting. The protocol was developed *a priori* but was retrospectively registered on the Open Science Framework (OSF) for transparency (https://doi.org/10.17605/OSF.IO/5SUDE).

### Search strategy

A comprehensive literature search was performed across four databases, PubMed, Scopus, Medline and CINAHL (accessed via EBSCOhost), to identify relevant studies published from 1 January 2015 to 12 May 2025. The same publication date range was applied across all databases. In Scopus, the year range 2015–2025 was used, which captured all records available up to the search date in May 2025. All searches were restricted to post-2014 publications to focus on recent AI advancements in machine learning, image recognition and related nutrition applications. The search strategy was designed to capture studies on AI technologies, mobile applications and UPF, including terms related to machine learning, image recognition and the NOVA classification system. A single search string was adapted for each database’s syntax, combining terms for AI and mobile apps with UPF-related terms:

- AI and mobile app terms: ‘artificial intelligence’, ‘machine learning’, ‘deep learning’, ‘neural network*’, ‘computer vision’, ‘image recognition’, ‘food recognition’, ‘natural language processing’, ‘automated dietary assessment’, ‘dietary assessment tool*’, ‘mobile application’, ‘app-based’, ‘smartphone application’.
- UPF terms: ‘ultra-processed food*’, ‘ultraprocessed food*’, ‘ultra processed food*’, ‘food processing’, ‘NOVA classification’, ‘ultra-processed product*’, ‘industrial food formulation*’, ‘food categorization’, ‘food categorisation’, ‘food classification’.
- Filters: Limited to English-language publications and studies published after 2014.

In PubMed, Medical Subject Headings (MeSH) and title/abstract ([tiab]) searches were used. Full search strategies are available in online Supplementary material. An example of the PubMed search is shown below:

((‘artificial intelligence’[MeSH Terms] OR ‘artificial intelligence’[tiab] OR ‘machine learning’[MeSH Terms] OR ‘machine learning’[tiab] OR ‘deep learning’[tiab] OR ‘neural network*’[tiab] OR ‘computer vision’[tiab] OR ‘image recognition’[tiab] OR ‘food recognition’[tiab] OR ‘natural language processing’[tiab] OR ‘automated dietary assessment’[tiab] OR ‘dietary assessment tool*’[tiab] OR ‘mobile application’[tiab] OR ‘app-based’[tiab] OR ‘smartphone application’[tiab])

AND

(‘ultra-processed food*’[tiab] OR ‘ultraprocessed food*’[tiab] OR ‘ultra processed food*’[tiab] OR ‘food processing’[tiab] OR ‘NOVA classification’[tiab] OR ‘IFIC classification’[tiab] OR ‘IFPRI classification’ [tiab] OR ‘SIGA classification’[tiab] OR ‘UNC classification’[tiab] OR ‘IARC classification’[tiab] OR ‘ultra-processed product*’[tiab] OR ‘industrial food formulation*’[tiab] OR ‘food categorization’[tiab] OR ‘food categorisation’[tiab] OR ‘food categorisation’[tiab] OR ‘food classification’[tiab])

AND

(‘2015/01/01’[Date - Publication] : ‘2025/05/12’[Date - Publication]))

In Scopus, title-abstract-keyword (TITLE-ABS-KEY) searches were applied, with additional filters to exclude book chapters and reviews. However, because many AI and computer science studies are published as peer-reviewed conference papers rather than traditional journal articles, these were retained when indexed as such (e.g. IEEE and Springer CCIS). This ensured that peer-reviewed conference proceedings were captured, while non-peer-reviewed book chapters were excluded. In EBSCOhost, searches were conducted in Medline and CINAHL, with title and abstract fields targeted and automatic deduplication applied by the platform. Search strings were tailored to each database’s syntax but maintained consistent terms. No grey literature or hand-searching was conducted at this stage. While this may have excluded some proprietary tools or unpublished implementations, the decision was made to prioritise studies providing sufficient methodological detail and peer-reviewed validation of AI approaches. This focus supports a more rigorous synthesis of evidence on performance, reproducibility and applicability of AI methods.

An updated search was conducted on 9 October 2025 following peer review to capture any newly indexed records that had not appeared at the time of the original search. Results were screened using the same inclusion and exclusion criteria as the original search, and one additional study was identified as within scope and incorporated into the synthesis. The updated search used the same databases, search terms and language limits as the original search; however, because databases do not allow retrospective filtering by date of indexing and Scopus does not permit day-level publication date filtering, it was not possible to generate a separate reproducible set of screening counts for the update. Because this update was undertaken to ensure currency rather than to re-run the full process, the PRISMA flow diagram remains based on the original search (ending May 2025), with newly identified records described narratively in the Results.

### Inclusion criteria

Studies were eligible for inclusion if they utilised or explicitly reported using AI-driven applications or tools, such as those employing machine learning, deep learning, computer vision, image recognition or NLP, to assess or classify the consumption or categorisation of UPF in the context of individual nutrition or population-level public health nutrition. Eligible studies categorised UPF using established classification systems (e.g. NOVA framework), emerging frameworks (e.g. HISS and IARC) or study-specific scales. Studies were also included if AI methods were applied to analyse patterns of UPF consumption or availability (e.g. dietary intake or food supply), provided that automated classification of foods by processing level formed an integral part of the analysis. Eligible studies targeted the general population of any age, region or setting for individual-level consumption assessments (e.g. dietary tracking via mobile apps or surveys) or broader applications (e.g. classifying supermarket foods or food supply inventories). Outcomes of interest included detailed reports on AI methodologies, performance metrics (e.g. accuracy and precision) and AI-driven classification results applied in contexts such as nutrition research, clinical practice, public health or consumer settings. The search was limited to articles published in English. Studies encompassed various designs, including observational and experimental studies, provided they contained methodological or empirical data.

### Exclusion criteria

Studies were excluded if they did not utilise AI, relying instead on manual dietary assessments or traditional statistical methods, or if they lacked a focus on UPF or food processing classification, such as those primarily concerned with quantifying nutrients or calorie counting without reference to processing levels. Additionally, studies were excluded if they addressed irrelevant contexts, such as AI applications in food production or agriculture (e.g. crop processing) not tied to consumption or classification. Studies were also excluded if AI or statistical methods were used solely to examine health outcomes (e.g. disease risk and biomarkers) using pre-coded NOVA variables, without analysing UPF classification or consumption patterns as a primary focus. Non-English publications and non-peer-reviewed sources like abstracts, editorials, opinion pieces or reviews lacking methodological or empirical detail were also excluded. Book chapters were excluded, as they generally do not undergo peer review or present primary empirical data. Studies focusing on animal diets or non-human populations were not considered.

### Data management

Search results were exported from PubMed, Scopus, and EBSCOhost as RIS files into EndNote for reference management. Records from each database were organised into separate groups to track their sources. Deduplication was performed using EndNote’s deduplication tool, followed by manual verification to ensure accuracy.

Title and abstract screening were conducted independently by JLC and HT, with discrepancies resolved through discussion. The same process was used for full-text screening and data extraction, with JLC primarily extracting data and HT independently reviewing all included studies to verify accuracy and consistency. Extraction focused on study characteristics such as design, AI methodology and the specific focus on UPF. Extracted information included author, year of publication, study aim and topic, key characteristics, findings, limitations and the type of AI method employed. Data were synthesised narratively to provide a comprehensive overview of the included studies. In line with the objectives of this scoping review, no formal critical appraisal of individual studies was undertaken.

## Results

### Identification of studies

**Figure 1.**
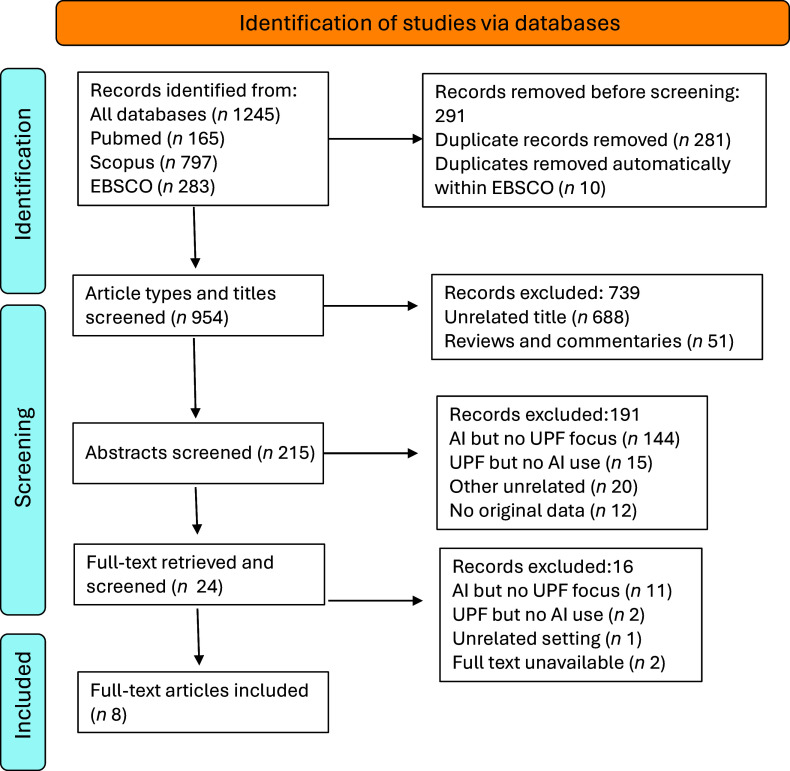
PRISMA-ScR flow diagram illustrating the process of study identification, screening, eligibility assessment and inclusion for the scoping review of AI applications in UPF assessment. This relates to the primary full systematic search conducted in May 2025. A brief update search was undertaken in October 2025 to identify any newly indexed studies and is reported narratively in the main text. AI, artificial intelligence; UPF, ultra-processed foods.

The primary systematic search yielded 1245 records across the three databases prior to deduplication (Figure 1). PubMed contributed 165 records, retrieved using MeSH terms and title/abstract searches. Scopus initially identified 1013 records which were further refined by excluding book chapters and reviews, resulting in 797 records, which were exported for screening. EBSCOhost (Medline and CINAHL) provided 283 records, which were reduced to 273 following automatic deduplication by the EBSCO platform. These 273 unique records were exported. In total, 1245 records (797 from Scopus, 165 from PubMed and 273 from EBSCOhost) underwent deduplication in EndNote, resulting in 954 unique records. Following title screening, 215 records remained, as many were excluded for focusing on irrelevant topics, such as disease detection, agricultural applications and chemical engineering (e.g. peptide analysis), or because they were reviews. After abstract screening, twenty-four records were retained for full-text review. Upon full-text evaluation, eight studies were included in the scoping review, as they fully met the eligibility criteria: Ase *et al.* 2025^(32)^, Elbassuoni *et al.* 2022^(33)^, Hu *et al.* 2023^(34)^, Menichetti *et al.* 2023^(35)^, Marcos *et al.* 2022^(36)^, Momanyi *et al.* 2025^(37)^, Ravandi *et al.* 2025^(38)^ and Solano *et al.* 2024^(39)^. These studies formed the basis for mapping the application of AI in assessing and classifying UPF. An identical updated search conducted in October 2025 to identify any newly indexed papers identified one additional eligible study (Arora *et al.* 2025^(40)^), which was incorporated into the synthesis but not reflected in the PRISMA flow diagram, as the update occurred *post hoc*.

### Overview of included studies

Among the nine included studies, six focused on classifying foods by processing level using large-scale datasets such as nutrient composition tables, ingredient lists or retail product data^(33–36,38,40)^. The remaining three studies explored consumption behaviours or practical applications of food processing classification in real-world or institutional settings^(32,37,39)^. Although the study by Momanyi *et al.*^(37)^ employed association rule mining, commonly classified as data mining rather than machine learning, it was retained due to its use of algorithmic methods on a large dataset (self-described as unsupervised learning), its population-level application and its explicit use of the NOVA classification framework. Table 1 provides a summary of key study characteristics, while Table 2 provides a high-level visual summary of similarities and differences between studies.

**Table 1.** Summary of included studies reporting AI methods for assessing and classifying UPF. The table shows country or setting, AI techniques, input data sources, dataset size, classification frameworks, outputs produced, application types and reported accuracy or validation measures

Study (year)	Country/setting	AI method	Input data	Sample size/dataset	Classification framework	Output	Application type	Accuracy/validation
Arora *et al.* (2025)	USA, national food databases	Supervised ML + DL + NLP (e.g. LGBM, Random Forest, Gradient Boost, SVM, logistic regression; NLP: BERT variants)	Nutrient profiles and textual features (descriptions, categories)	2970 food items from USDA FNDDS 2009–2010	NOVA	Predicted NOVA category per food item	Automated food processing classification; public health monitoring; web server for predictions	F1 = 0·96; MCC = 0·91 (NLP) and F1 = 0·94; MCC = 0·87 (nutrient-only)
Ase *et al.* (2025)	Poland, long-term care facilities; food items sourced from resident cabinet inventories via interviews	LLM Claude 3.7 Sonnet – an advanced AI model	Unstructured product names/descriptions (interview-based; no nutrition label or ingredient data)	1543 cabinet food products from 693 residents	NOVA + WHO (two-step, first NOVA then non-UPF analysed via WHO criteria for sugars, saturated fat and Na).	Binary classification: ‘healthy’ *v*. ‘unhealthy’ foods; 15·6 % classified as ‘unhealthy’ foods.	Institutional dietary risk assessment	Accuracy of AI not reported or verified
Elbassuoni *et al.* (2022)	Tunisia, real-world food image dataset from wearable cameras	Deep learning (YOLOv3 object detection and MobileNetV2 classification)	Images of Tunisian foods	3728 labelled food and beverage item images	NOVA	NOVA categories	Image-based dietary assessment; potential for real-time mobile application	NOVA classifier: F1 = 0·86 (on test set)
Hu *et al.* (2023)	Canada, Argentina, USA, national food composition databases	Fine-tuned LLM (used BERT)	Ingredient lists; also tested nutrient data and bag of words	Training on 18 916 products (Canada, 2017); validated on 74 445 (Canada, 2020), 8465 (Argentina) and 1·7M (USA)	NOVA	Nova category	Food supply-level UPF classification	F1 score = 0·979 (Canada, 2017); generalised to datasets from Argentina (0·889) and the USA (0·947)
Menichetti *et al.* (2023)	USA, national food supply	Random forest classifier (FoodProX)	Nutrient profiles of packaged foods, additional model tested with additive presence from Open Food Facts	7253 US food items (used for model training). 233 831 packaged foods (external validation). 20 047 adult dietary records (population-level application)	NOVA and NOVA-derived FPro score (continuous index)	Food level: NOVA category + FPro score (0–1 scale) Individual-level: iFPro (calorie- and gram-weighted processing scores) Health correlations: metabolic risk, vitamin levels, chemical exposure	National food supply classification Individual diet profiling (via NHANES) Public health risk modelling and substitution analysis	FPro predicts NOVA with > 95 % accuracy across food categories FPro score stable despite large changes in nutrient inputs Validated against population health data and external food databases
Marcos *et al.* (2022)	USA, national food database	Unsupervised learning (e.g. Gaussian mixture, k-means, spectral)	Nutrient profiles: three datasets tested with increasing feature complexity (12, 62 and 99 variables)	2917 food items across NOVA groups 1, 3 and 4	NOVA	Cluster membership and level of alignment with NOVA group labels	National food supply classification	Gini coefficient ranges from 0·21 to 0·54; best performance from the Gaussian mixture model on the mid-size dataset (62 features)
Momanyi *et al.* (2025)	Kenya, supermarkets	Data mining (*a priori* algorithm); described as unsupervised machine learning	Grocery purchase data	Data from 103 931 shoppers (> 3·9 million transactions)	NOVA	NOVA category distribution (e.g. % UPF)	Purchase pattern analysis across demographics	53 % of purchases classified as UPF; no formal model validation or performance metrics reported
Ravandi *et al.* (2025)	USA, grocery database from three supermarkets	Machine learning (FPro)	Ingredient lists and nutrient labels	Over 50 000 food products	FPro (derived from NOVA)	FPro score (0–1) per food; supermarket-level processing distribution; substitution suggestions for less processed alternatives	Product-level classification; consumer decision support; supermarket-level food environment analysis	Validated on NHANES and other datasets; FPro scores range from 0 to 1, strong correlation with NOVA labels
Solano *et al.* (2024)	Ecuador, general population; focus on T2D prevention	Machine learning for food clustering; chatbot uses an LLM	Nutrient composition of Ecuadorian foods; supermarket product data; knowledge graph (user + dietary info)	> 1000 foods from national nutrient databases and INFORMAS-style supermarket audit	NOVA	Substitute food recommendations for NOVA 3/4 products; binary chatbot responses	Mobile substitution app + nutrition chatbot for T2D risk reduction	Expert review of 318 substitute pairs: about 58 % accuracy, 93 % relevancy, 55 % appropriateness (higher for snacks, lower for mixed dishes)

UPF, ultra-processed foods; ML, machine learning; DL, deep learning; NLP, natural language processing; LGBM, light gradient boosting machine; SVM, support vector machine; BERT, Bidirectional Encoder Representations from Transformers; FNDDS, Food and Nutrient Database for Dietary Studies; F1, F1 score (harmonic mean of precision and recall); LLM, large language model; YOLOv3, You Only Look Once (version 3); FPro, iFPro, Food Processing/individual Food Processing score; NHANES, National Health and Nutrition Examination Survey; USDA, United States Department of Agriculture; T2D, type 2 diabetes; MCC, Matthews Correlation Coefficient.

**Table 2.** High-level summary showing similarities and differences between the included studies

	AI method			Input data						
Study	Supervised learning	Unsupervised learning	Rule-based/hybrid	Nutrient data	Ingredient/text input	Image input	Purchase/behavioural data	Classification	Accuracy reported	Real-world application/context^*^
Arora *et al.* 2025	✓			✓	✓			NOVA	✓	
Ase *et al.* 2025			✓		✓		✓	NOVA + WHO		✓
Elbassuoni *et al.* 2022	✓					✓		NOVA	✓	
Hu *et al.* 2023	✓			✓	✓			NOVA	✓	
Marcos *et al.* 2022		✓		✓				NOVA	Cluster alignment	
Menichetti *et al.* 2023	✓			✓				NOVA (FPro)	✓	
Momanyi *et al.* 2025		(Self-described)	✓				✓	NOVA		✓
Ravandi *et al.* 2025	✓			✓				NOVA (FPro)	✓	✓
Solano 2024		✓	✓	✓				NOVA	Expert review	✓

AI, artificial intelligence; FPro, Food Processing; UPF, ultra-processed foods; NHANES, National Health and Nutrition Examination Survey.

* Real-world application/context refers to studies in which AI-based UPF classifications were applied within practical settings or datasets derived from real environments (e.g. supermarket purchasing data and institutional food audits) or where a prototype tool was deployed and evaluated (e.g. usability testing). Analytical modelling studies, including those involving public-facing tools that were not used, evaluated or deployed in the study itself, were not classified as real-world applications. For example, Menichetti *et al.* (2023)^(35)^ provided a publicly accessible FPro calculator but did not assess its use, performance or behavioural impact in consumer or clinical contexts; their analysis was limited to computational modelling using NHANES dietary records.

### Artificial intelligence methods used

The included studies employed a diverse range of AI and computational techniques to classify or analyse food processing levels. These fell broadly into three categories: supervised machine learning, unsupervised learning and rule-based logic systems.

Supervised models were most common. Two studies^(35,38)^ used FoodProX, a random forest classifier trained to predict NOVA categories based on nutrient profiles. Hu *et al.*^(34)^ developed a transformer-based language model (fine-tuned Bidirectional Encoder Representations from Transformers (BERT)) to classify foods using ingredient list text. Arora *et al.*^(40)^ also developed and compared multiple supervised and NLP models, including logistic regression, random forest, support vector machine and transformer-based approaches, to predict NOVA categories from nutrient profiles and textual features. Elbassuoni *et al.* (2022) developed a deep learning image classifier based on a convolutional neural network trained to assign NOVA levels to foods in photographs.

Unsupervised methods were also used. Marcos *et al.*^(36)^ applied unsupervised clustering techniques (e.g. k-means) to nutritional data from national food databases, using NOVA only for cluster validation. Momanyi *et al.*^(37)^ used an unsupervised learning approach, *a priori* association rule mining, to explore purchasing patterns, applying predefined NOVA categories to food items. Solano *et al.*^(39)^ similarly employed unsupervised clustering (Self-Organizing Map with K-means) to group foods by nutrient similarity, forming the basis of a hybrid system (described below).

Finally, rule-based approaches were used in two studies. Ase *et al.*^(32)^ employed a proprietary large language model (Claude 3.7 Sonnet) to interpret free-text food descriptions and support a rule-based classification system. Solano *et al.*^(39)^ integrated rule-based logic with unsupervised clustering outputs to classify foods by NOVA level and nutrient cut-offs, powering a substitution tool that recommended healthier alternatives.

### Input data and classification frameworks

Input data varied widely across the studies, encompassing structured datasets (e.g. ingredient lists, nutrient panels and food images) as well as unstructured or behavioural data (e.g. food descriptions or purchase records).

Seven studies^(33–36,38–40)^ relied primarily on structured product-level data from food composition databases, retail product information or labelled image repositories. For example, Hu *et al.*^(34)^ used ingredient list text from national food product databases, while Elbassuoni *et al.*^(33)^ trained and tested their model on over 14 000 NOVA-labelled food images, including images captured via wearable cameras and curated datasets. Arora *et al.*^(40)^ used nutrient profiles and textual features from USDA Food and Nutrient Database for Dietary Studies (FNDDS) items to train language models for automatic NOVA classification. Marcos *et al.*^(36)^ analysed FNDDS and recipe nutrient composition data to infer natural food clusters, and Solano *et al.*^(39)^ leveraged datasets from previous nutritional studies to build a substitution tool grounded in nutrient thresholds and NOVA classification.

In contrast, Ase *et al.*^(32)^ used qualitative food descriptions collected via interviews with long-term care facility residents and caregivers, while Momanyi *et al.*^(37)^ used supermarket loyalty card transaction records linked to customer demographic data.

All studies applied the NOVA framework to define food processing levels. Two studies, Menichetti *et al.*^(35)^ and Ravandi *et al.*^(38)^, extended NOVA by generating continuous scores (FPro) to represent processing on a granular scale. Ase *et al.*^(32)^ applied a two-step system: initial NOVA categorisation followed by classification using WHO thresholds for Na, sugar and saturated fat, to assign a binary ‘healthy’ or ‘unhealthy’ label. Momanyi *et al.*^(37)^ directly applied NOVA categories to retail product data for downstream analysis, Elbassuoni *et al.*^(33)^ used NOVA as ground truth for image classification, Marcos *et al.*^(36)^ assessed cluster alignment with NOVA levels, Arora *et al.*^(40)^ trained multiple supervised models using NOVA-labelled datasets as ground truth and Solano *et al.*^(39)^ combined NOVA with nutrient profiling to evaluate substitution outcomes.

### Classification outputs

All studies generated food-level classification outputs, with several also producing higher-order tools or individual-level metrics.

Hu *et al.*^(34)^, Menichetti *et al.*^(35)^ and Ravandi *et al.*^(38)^ produced large-scale classifications across national or commercial food datasets using either the NOVA framework or its continuous derivative, FPro. Arora *et al.*^(40)^ generated large-scale automated NOVA classifications using nutrient profiles and textual features, demonstrating that NLP models can effectively replicate manual classification processes. Menichetti *et al.*^(35)^ further developed an individual dietary score (iFPro) to estimate UPF exposure, which was applied in secondary analyses linking processing levels to health outcomes using National Health and Nutrition Examination Survey (NHANES) data. Ravandi *et al.*^(38)^ applied FPro to over 50 000 supermarket products, comparing processing levels across retailers and product categories and built a substitution tool suggesting less processed alternatives.

Marcos *et al.*^(36)^ used clustering techniques to infer natural food groupings from nutrient data, assigning NOVA labels *post hoc* for interpretability, and Elbassuoni *et al.*^(33)^ generated NOVA-level predictions from food images, enabling automated dietary classification from photos. Ase *et al.*^(32)^ implemented a binary ‘healthy’/‘unhealthy’ labelling system based on a combined NOVA-WHO approach; while AI classification was a core output, their primary analysis focused on demographic and physical correlates of unhealthy food intake. Momanyi *et al.*^(37)^ classified retail food purchases by NOVA level, reporting population-level UPF proportions and demographic consumption patterns. Solano *et al.*^(39)^ developed a dynamic substitution tool using rule-based comparisons of NOVA level and nutrient thresholds.

### Application contexts

The studies applied AI-driven food classification across a variety of analytical and practical settings, with most conducted in high-income countries.

Five studies^(34–36,38,40)^ focused on characterising food environments in North America. Hu *et al.*^(34)^ classified foods in national branded product databases from Canada, the USA and Argentina. Menichetti *et al.*^(35)^ applied iFPro scores to individual dietary records from NHANES to examine health associations, while Ravandi *et al.*^(38)^ assessed processing levels across major US retailers (e.g. Walmart, Target and Whole Foods), developing a substitution tool and comparing UPF prevalence by category and price. Arora *et al.*^(40)^ conducted a methodological comparison of supervised and NLP-based models using datasets from US food products to enhance the scalability and reproducibility of NOVA classification. Marcos *et al.*^(36)^ conducted a methodological exploration using unsupervised clustering of nutrient profiles from US food composition databases, with the goal of informing food surveillance and reformulation policies.

Other studies were conducted in institutional or emerging settings. Ase *et al.*^(32)^ audited cabinet foods in long-term care facilities in Poland, using AI-driven NOVA and nutrient threshold classifications to examine associations with residents’ demographic and physical characteristics. Momanyi *et al.*^(37)^ analysed supermarket transaction data in Kenya, applying NOVA classifications to explore demographic drivers of UPF purchases by age, sex and region, offering one of the few perspectives from a sub-Saharan African retail context. Solano *et al.*^(39)^, based in Ecuador, developed a rule-based substitution tool for use in diabetes prevention and public health nutrition, proposing its application in clinical and consumer-facing dietary guidance. Elbassuoni *et al.*^(33)^ trained their food image classification model on a dataset including Tunisian food images and curated global datasets, positioning their system for passive dietary monitoring in free-living settings via wearable cameras. Across the included studies, four were applied in practical or real-world contexts: two^(32,37)^ examined consumption or purchasing behaviours, and two^(38,39)^ developed substitution tools to support healthier choices in retail and consumer settings.

### Accuracy and validation

Most studies reported formal accuracy or performance metrics for their AI models. Hu *et al.*^(34)^ achieved excellent predictive accuracy in classifying NOVA levels across three national food datasets (F1 = 0·979 for Canadian foods), with similar performance in US and Argentinian products. The F1 score is a standard metric in machine learning that balances precision (how many predicted positives are correct) and recall (how many actual positives were identified), offering a single measure of overall model performance. Menichetti *et al.*^(35)^ reported high classification performance for FoodProX, with AUC values exceeding 0·95 across all NOVA categories, and found significant correlations between iFPro scores and health outcomes in NHANES. Ravandi *et al.*^(38)^ reported high predictive accuracy of FPro scores when benchmarked against expert NOVA labels, achieving classification consistency above 90 % across food categories. Arora *et al.*^(40)^ reported comparable model accuracy across several supervised and NLP approaches, confirming strong predictive capacity from nutrient profiles and textual features.

Elbassuoni *et al.*^(33)^ reported classification accuracy above 90 % on an external test set of labelled food images. Marcos *et al.*^(36)^, using unsupervised clustering, did not report predictive metrics but demonstrated strong alignment between emergent clusters and NOVA groups, supporting construct validity.

Three studies, Ase *et al.*^(32)^, Momanyi *et al.*^(37)^ and Solano *et al.*^(39)^, did not report formal performance metrics. The analyses by Ase *et al.*^(32)^ and Momanyi *et al.*^(37)^ were exploratory in nature, applying classification outputs to population or behavioural analyses. The tools used by Solano *et al.*^(39)^ relied on predefined logic rules rather than a predictive model, precluding traditional performance evaluation.

## Discussion

This scoping review identified nine peer-reviewed studies applying AI methods to the classification or assessment of UPF. Although their design, data sources and analytical approaches varied, all studies relied on the NOVA framework or derived adaptations to define processing levels. Techniques ranged from supervised learning and NLP to rule-based approaches, applied to diverse datasets including nutrient profiles, product information and behavioural records. Applications encompassed food environment profiling, institutional dietary audits and population-level dietary analysis.

### Significance of the evidence gap

Although only nine studies met the inclusion criteria for this review, this limited yield is itself a meaningful finding. It highlights a notable gap at the intersection of two rapidly advancing domains: AI in dietary assessment and the growing body of literature on UPF. Over the past decade, interest in UPF has grown rapidly, with thousands of studies examining their association with metabolic health, dietary quality and disease risk^(8,9,12,13,15)^. Concurrently, AI has been increasingly applied to nutrition science, particularly in the development of tools for estimating energy and nutrient intake, food identification, portion size estimation and behavioural monitoring^(26,28–30)^.

Recent reviews on AI use in nutrition including systematic^(26)^ and scoping reviews^(27,28,30)^ collectively screened over 8000 records and identified between 22 and 84 studies each, illustrating the rapid growth of AI applications in nutrition research. In contrast, the current review identified nine studies at the intersection of AI and food processing classification, highlighting how little attention this specific area has received despite broader interest in AI-driven dietary assessment. This disconnect is particularly striking given the prominence of UPF in recent dietary guidelines and public health discourse; a 2023 bibliometric analysis identified 1018 publications on UPF between 2010 and 2022, with over 70 % published in the last 6 years^(15)^. The expanding literature is also evident in the rising number of narrative and systematic reviews, meta-analyses and policy discussions engaging with the topic of UPF.

Notably, however, two of the included studies, Ravandi *et al.*^(38)^ and Menichetti *et al.*^(35)^, encompassed multiple methodological and applied components, combining the development of new processing metrics with applications in dietary profiling and consumer tools. These multifaceted contributions suggest that the body of relevant work may be larger than the publication count implies. Further, Arora *et al.*^(40)^ recently expanded the work of Menichetti *et al.*^(35)^ by applying additional machine learning and NLP approaches to nutrient-based classification, while a preprint by Kondaparthy *et al.*^(41)^ (not part of the included studies) demonstrated an integrated NOVA and Nutri-Score prediction pipeline. This suggests that while initially the area was slower to progress, it is an emerging field. Nevertheless, the small number of eligible studies reinforces the significance of this scoping effort in identifying a clear gap in the literature and highlighting a critical area for future research and tool development.

### Artificial intelligence methods and classification frameworks

The included studies demonstrated methodological innovation in applying AI approaches to UPF classification. Collectively, they leveraged diverse machine learning and NLP techniques, including comparing multiple model architectures for nutrient profiles and textual features-based classification^(40)^, alongside large-scale datasets. This illustrates the potential of AI to enhance measurement precision, reduce bias and enable scalable dietary surveillance, as noted by Morgenstern *et al.*^(42)^. Technical performance was generally robust, with several models achieving high predictive accuracy. Importantly, some studies also applied these methods in under-represented populations, demonstrating AI’s feasibility across diverse cultural and socio-economic contexts.

All studies used the NOVA classification framework, the most widely applied system in public health nutrition, to define food processing levels. However, NOVA’s reliance on qualitative criteria and limited differentiation within categories has sparked debate. To address this, two studies^(35,38)^ developed a continuous processing score offering a more granular and reproducible index. Similarly, Ase *et al.*^(32)^ implemented a two-step classification, combining NOVA with WHO nutrient thresholds for Na, sugar and saturated fat to assess healthfulness. Nevertheless, it was evident that in the majority of cases, accuracy and validity were being determined by the fidelity to NOVA. This in itself is something that we believe warrants discussion. This point is particularly relevant for interpreting the included studies, as the reliance on NOVA as a benchmark underpins both the consistency and limitations observed across the evidence base. Treating NOVA as an unquestioned gold standard conflates reproducibility with correctness, and divergence from NOVA classifications may reflect limitations of the framework itself rather than deficiencies in alternative or data-driven approaches. Despite widespread criticism over the past decade^(21,43–47)^, NOVA has evolved little, and its conceptual ambiguities remain unresolved. For instance, the FPro-based website frequently identifies plant-based milks as highly processed, recommending less processed alternatives that are predominantly cows’ milk, with occasional plant-based options among the suggestions. This raises questions about defining ‘healthier’ when dietary patterns, ethical preferences or broader health impacts are not adequately integrated. Moreover, there is a risk of conflating the availability of nutrient composition data with a meaningful assessment of processing, as Marcos and colleagues^(36)^ have noted that robust classification requires at least twenty nutritional parameters for model training – far more than food manufacturers are required to disclose in most jurisdictions.

Another notable development is that much of this work is now being undertaken by computer science departments. While these groups bring valuable technical expertise, they often lack grounding in nutrition science and dietary contexts. This may contribute to the uncritical adoption of NOVA as the benchmark, with limited awareness of the long-standing controversies surrounding its use. For example, in one recent publication, turmeric and saffron were mistakenly placed in inappropriate NOVA categories^(36)^, a detail that, while not invalidating the technical aspects of the work, highlights the importance of interdisciplinary collaboration to ensure both methodological rigour and domain relevance. Recent contributions by Arora *et al.*^(40)^ and Kondaparthy *et al.*^(41)^ further exemplify this trend: both studies achieved strong technical performance but relied on NOVA labels as ground truth without critically addressing the framework’s conceptual limitations. Relatedly, we also observed during the screening process that many studies, often originating from computer science fields, attempted to classify foods as ‘healthy’, ‘unhealthy’ or ‘junk food’ or give nutritional advice, without providing clear data sources or rationale for how these groupings were defined^(48–50)^. While these efforts represent important steps in applying computational methods to nutrition, they also illustrate the need for transparent criteria and stronger links between computational methods and nutritional evidence.

### Limitations in the evidence base

Despite promising developments, the evidence base for AI applications in UPF classification remains in its early stages. Several included studies were exploratory, with limited validation in real-world dietary intake contexts. One study^(33)^ used image-based classification, and only one, Solano *et al.*^(39)^, implemented a mobile application. However, none were designed to quantify daily UPF intake or deliver real-time dietary feedback, despite the growing role of both in nutrition tracking and behaviour change. While the FPro scoring system is publicly accessible online, only Solano *et al.*^(39)^ evaluated user experience through usability testing and expert verification of substitution outputs. One study^(35)^ conducted isoenergetic substitution modelling using NHANES dietary records; however, this represented simulated nutrient-based replacements rather than real-time consumer interaction or behavioural assessment. However, no included studies assessed the real-world impact, interpretability or behavioural consequences of AI-generated UPF classifications in consumer or clinical contexts. Broader deployment of AI-based classifiers in consumer settings, particularly those involving real-time dietary feedback, remains a critical gap. Developing tools for low-resource settings, where UPF prevalence is increasing, is also essential for equitable public health impact.

### Implications for research and practice

This review highlights a clear gap in the application of AI specifically for UPF assessment. Future research should prioritise the development of transparent, standardised AI models capable of reliably classifying foods by processing level across diverse food environments. There are also opportunities for the development of tools that integrate multiple AI techniques, such as image recognition, NLP and rule-based systems, for real-world dietary assessment and public health surveillance. Importantly, further studies should continue to extend beyond high-income settings to include culturally and economically diverse contexts, ensuring global applicability and equity in AI-assisted dietary assessment. Ongoing research is addressing these gaps, including a mobile application developed by members of this research team that uses computer vision to classify foods by processing level via the HISS framework and estimates the proportion of daily UPF intake^(51,52)^. This work reflects the potential of integrated AI systems to support both real-time dietary feedback and population-level monitoring of UPF consumption. Beyond NOVA, future work should also explore alternative or complementary classification systems (such as SIGA, IFIC, IARC or HISS), which offer differing conceptualisations of food processing, from nutrient profiling integration to product formulation scoring, and may enhance the granularity, reproducibility and cultural adaptability of AI-based classifications. These advancements could ultimately inform policy by enabling precise UPF monitoring for dietary guidelines and interventions.

### Strengths and limitations of the review

This scoping review has several notable strengths. It is the first to specifically examine the application of AI methods for assessing and classifying UPF consumption, addressing a growing area of public health and nutrition technology. A comprehensive and systematic search strategy was applied across four major databases (PubMed, Scopus, Medline and CINAHL), ensuring broad coverage of the literature. Predefined eligibility criteria were applied consistently, and the inclusion of diverse AI methodologies ranging from NLP and large language models to rule-based approaches provides a rich overview of current approaches in the field. The review also captures applications across multiple settings, including consumer, research and public health contexts.

However, several limitations should be noted. The number of included studies was small, reflecting the early stage of this research area. Grey literature and proprietary tools were excluded, which may have omitted relevant but unpublished or commercial applications of AI for UPF assessment. The review was limited to English-language publications, introducing potential language bias. Finally, no studies systematically evaluated the usability or interpretability of AI-generated classifications from the perspective of end users, such as consumers or health professionals, though Solano *et al.*^(39)^ conducted expert verification of substitution outputs.

### Conclusions

This scoping review represents the first systematic effort to map the application of AI in assessing and classifying UPF consumption, illuminating a nascent yet critical intersection of nutrition science and technology. The identification of only nine eligible studies highlights a significant gap in the application of AI for UPF assessment, despite the rapid expansion of both AI-driven dietary tools and UPF-related research. Future research is essential to develop robust, transparent and globally relevant AI models that integrate advanced methodologies, such as computer vision and NLP, to classify foods by processing level in real-world contexts. Extending investigations to diverse socio-economic and cultural settings, coupled with rigorous validation of consumer-oriented tools, will be crucial to inform public health strategies aimed at reducing UPF consumption. Rather than a limitation, we suggest that the limited number of studies is itself a key finding, highlighting a pressing opportunity for innovation in AI-driven nutrition assessment to address the global proliferation of UPF.

## Supporting information

Campbell et al. supplementary material Campbell et al. supplementary material

## Supplementary material

For supplementary material/s referred to in this article, please visit https://doi.org/10.1017/S000711452510593X
