# Supplementary material for: Artificial intelligence applications for assessing ultra-processed food consumption: a scoping review
Source: Br J Nutr. 2025 Dec 22;135(4):463–73. doi: 10.1017/S000711452510593X (PMC12929014; doi:10.1017/S000711452510593X)
Supplement: Campbell et al. supplementary material [file S000711452510593Xsup001.docx]

**Supplementary material - Search strings**

**PubMed**

(("artificial intelligence"[MeSH Terms] OR "artificial intelligence"[tiab] OR "machine learning"[MeSH Terms] OR "machine learning"[tiab] OR "deep learning"[tiab] OR "neural network*"[tiab] OR "computer vision"[tiab] OR "image recognition"[tiab] OR "food recognition"[tiab] OR "natural language processing"[tiab] OR "automated dietary assessment"[tiab] OR "dietary assessment tool*"[tiab] OR "mobile application"[tiab] OR "app-based"[tiab] OR "smartphone application"[tiab])

AND

("ultra-processed food*"[tiab] OR "ultraprocessed food*"[tiab] OR "ultra processed food*"[tiab] OR "food processing"[tiab] OR "NOVA classification"[tiab] OR " IFPRI classification"[tiab] OR " IFIC classification"[tiab] OR " IARC classification"[tiab] OR "SIGA classification"[tiab] OR "UNC classification"[tiab] OR "EPIC classification"[tiab] OR "ultra-processed product*"[tiab] OR "industrial food formulation*"[tiab] OR "food categorization"[tiab] OR "food categorisation"[tiab] OR "food classification"[tiab])

AND

("01/01/2015"[Date - Publication] : " 05/12/2025"[Date - Publication]))

*Displayed as (MM/DD/YYYY)

**Scopus**
TITLE-ABS-KEY ( "artificial intelligence" OR "machine learning" OR "deep learning" OR "neural network*" OR "computer vision" OR "image recognition" OR "food recognition" OR "natural language processing" OR "automated dietary assessment" OR "dietary assessment tool*" OR "mobile application" OR "app-based" OR "smartphone application" ) AND TITLE-ABS-KEY ( "ultra-processed food*" OR "ultraprocessed food*" OR "ultra processed food*" OR "food processing" OR "NOVA classification" OR "ultra-processed product*" OR "IFIC classification" OR " IFPRI classification" OR " IARC classification" OR "SIGA classification" OR "UNC classification" OR "EPIC classification” OR "industrial food formulation*" OR "food categorization" OR "food categorisation" OR "food classification" )

Filtered by year (including only papers after 2014), and filtered by excluding books, book chapters and reviews. Since the search was conducted in May 2025, this effectively set a cut off date of 12^th^ May, inline with EBSCO and Pubmed.

**CINAHL and MedLine (via EBSCO)**

Date range = 01/01/2015 – 12/05/2025

*Displayed as (DD/MM/YYYY)

English language and academic journals

Advanced search (all fields):

( "artificial intelligence" OR "machine learning" OR "deep learning" OR "neural network*" OR "computer vision" OR "image recognition" OR "food recognition" OR "natural language processing" OR "automated dietary assessment" OR "dietary assessment tool*" OR "mobile application" OR "app-based" OR "smartphone application" )

AND

( "ultra-processed food*" OR "ultraprocessed food*" OR "ultra processed food*" OR "food processing" OR "NOVA classification" OR "IFIC classification" OR " IFPRI classification" OR " IARC classification" OR "SIGA classification" OR "UNC classification" OR "EPIC classification” OR "ultra-processed product*" OR "industrial food formulation*" OR "food categorization" OR "food categorisation" OR "food classification" )
